# P-837. Effect of Clinical Guideline Implementation on Use of Plasma Microbial Cell-free DNA Sequencing Testing at an Academic Medical Center

**DOI:** 10.1093/ofid/ofaf695.1045

**Published:** 2026-01-11

**Authors:** Trevor C Van Schooneveld, Tess Karre, Jonathan H Ryder

**Affiliations:** University of Nebraska Medical Center, Omaha, NE; University of Nebraska Medical Center, Omaha, NE; University of Nebraska Medical Center, Omaha, NE

## Abstract

**Background:**

The role and timing of plasma microbial cell-free DNA (mcfDNA) testing is unknown. We created a guideline for use arranged by clinical syndrome and evaluated changes in testing, results, and impact on patient care pre- and post-guideline.

**Table 1:**
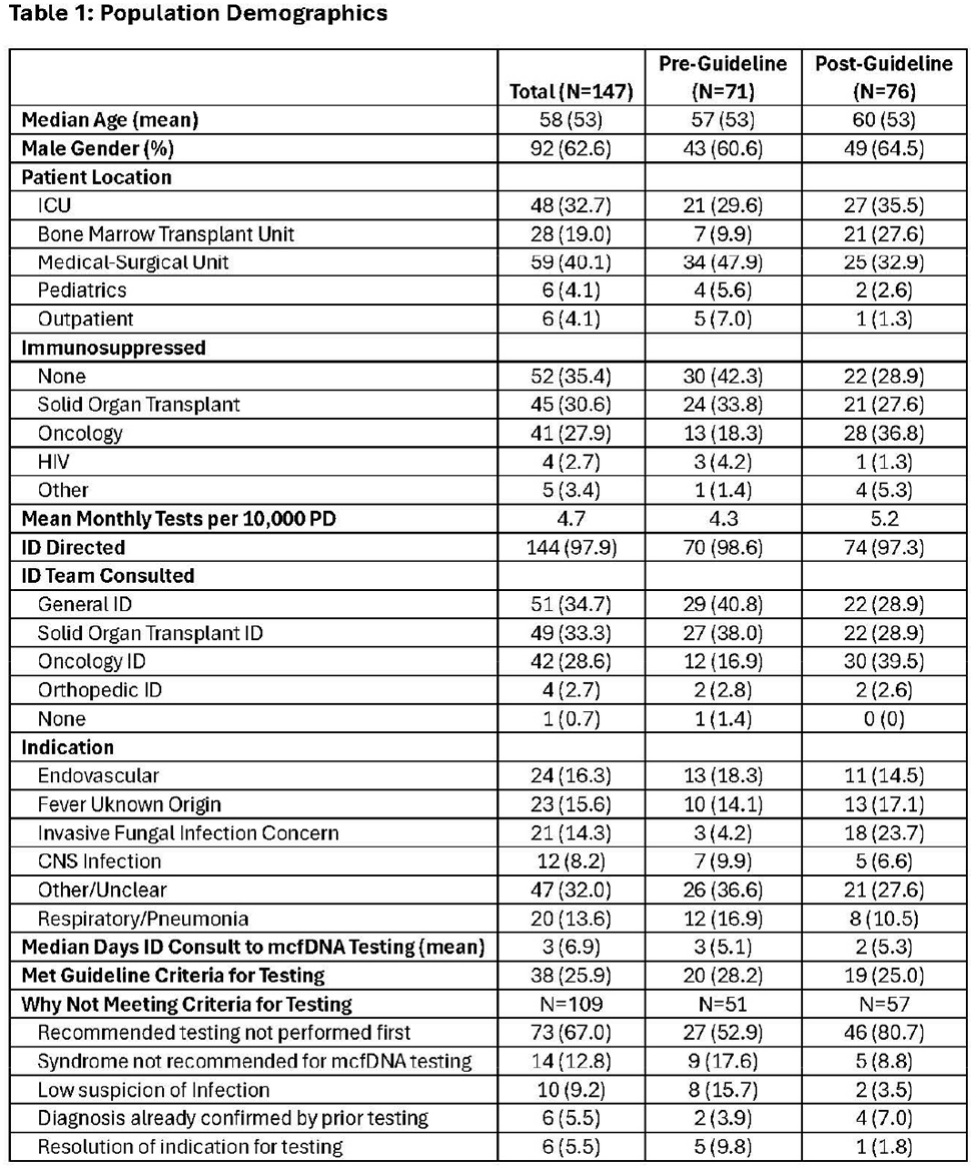
Population Demographics

**Table 2:**
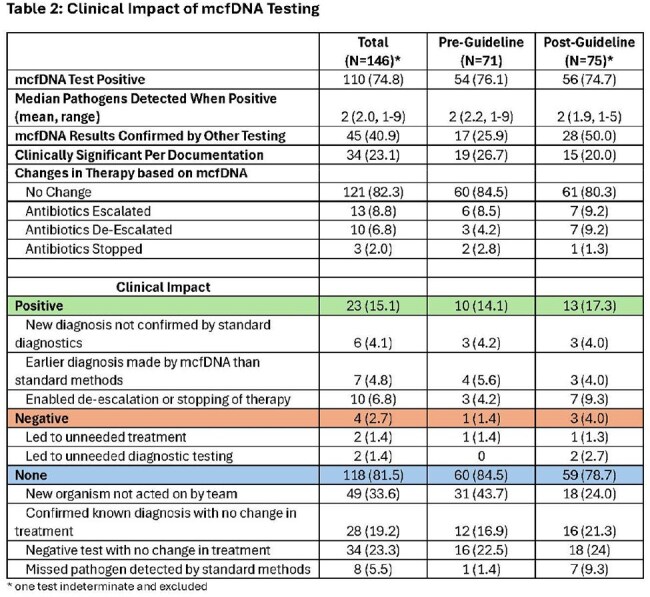
Clinical Impact of mcfDNA Testing

**Methods:**

The stewardship program created a clinical guideline with input from microbiology and ID providers regarding use and timing of mcfDNA testing. It was published online 3/2024 with accompanying brief education to ID. We compared mcfDNA testing pre- (4/2023-2/2024) and post-guideline (3/2024-12/2024). Testing appropriateness was assessed based on institutional guidance. Indication for testing, clinical significance and impact was determined using clinical documentation.

**Results:**

A total of 147 mcfDNA tests were ordered on 142 patients with demographics in Table 1. Most tests were in immunocompromised patients. Testing increased slightly post-guideline (4.3 vs. 5.2 tests/10000 PD). mcfDNA tests were positive in 75% tested for a median of 2 pathogens with at least some results confirmed by other testing in 41% (Table 2). Post-guideline testing for Other and Respiratory diagnoses decreased but testing by Oncology ID increased (16.9% vs. 39.5%) with a 6-fold increase in use for suspected Invasive Fungal Infection (IFI) (4.2% vs. 23.7%). Only 23% of mcfDNA tests were considered clinically significant by providers (pre- 26.7% vs. 20.0% post-).

Overall, 25.9% (38/147) of tests were judged appropriate with no change pre- vs. post-guideline (28.2% vs. 25.0%, Table 1). The most common reason for not meeting testing criteria was recommended testing not performed first and this increased post-guideline (52.9% vs. 80.7%). Changes to therapy were uncommon based on mcfDNA and did not change much post-guideline (Table 2). Patient care impact did not change either with 81.5% of all results having no impact, 15.1% positive, and 2.7% negative.

**Conclusion:**

mcfDNA testing was relatively low yield, usually ordered before other testing had been performed/resulted, and exhibited no change in appropriateness or yield after guideline publication. This data will be used to educate ID providers on effective use strategies as well as assist in potential guideline revision.

**Disclosures:**

Trevor C. Van Schooneveld, MD, FSHEA, FIDSA, BioMerieux: Advisor/Consultant|BioMerieux: Grant/Research Support

